# Exploration of the Gut–Brain Axis through Metabolomics Identifies Serum Propionic Acid Associated with Higher Cognitive Decline in Older Persons

**DOI:** 10.3390/nu14214688

**Published:** 2022-11-05

**Authors:** Jeanne Neuffer, Raúl González-Domínguez, Sophie Lefèvre-Arbogast, Dorrain Y. Low, Bénédicte Driollet, Catherine Helmer, Andrea Du Preez, Chiara de Lucia, Silvie R. Ruigrok, Barbara Altendorfer, Ludwig Aigner, Paul J. Lucassen, Aniko Korosi, Sandrine Thuret, Claudine Manach, Mercè Pallàs, Mireia Urpi-Sardà, Alex Sánchez-Pla, Cristina Andres-Lacueva, Cécilia Samieri

**Affiliations:** 1 Bordeaux Population Health Research Center, University of Bordeaux, INSERMUMR 1219, F-33000 Bordeaux, France; 2 Nutrition, Food Science and Gastronomy Department, Food Innovation Network (XIA), Institute of Nutrition and Food Safety (INSA-UB), Faculty of Pharmacy and Food Science, University of Barcelona, 08028 Barcelona, Spain; 3 CIBER Fragilidad y Envejecimiento Saludable (CIBERFES), Instituto de Salud Carlos III, 28029 Madrid, Spain; 4 Human Nutrition Unit, Université Clermont Auvergne, INRAEUMR1019, F-63000 Clermont Ferrand, France; 5 Lee Kong Chian School of Medicine, Nanyang Technological University, Singapore 636921, Singapore; 6 Department of Basic and Clinical Neuroscience, Maurice Wohl Clinical Neuroscience Institute, Institute of Psychiatry, Psychology and Neuroscience, King’s College London, London SE5 9NU, UK; 7 Brain Plasticity Group, Swammerdam Institute for Life Sciences, University of Amsterdam, 1098 XH Amsterdam, The Netherlands; 8 Institute of Molecular Regenerative Medicine, Spinal Cord Injury and Tissue Regeneration Center Salzburg, Paracelsus Medical University, 5020 Salzburg, Austria; 9 The Center for Urban Mental Health, University of Amsterdam, 1098 XH Amsterdam, The Netherlands; 10 Department of Neurology, University Hospital Carl Gustav Carus, Technische Universität Dresden, 01307 Dresden, Germany; 11 Pharmacology Section, Department of Pharmacology, Toxicology and Medicinal Chemistry, Faculty of Pharmacy and Food Sciences, and Institute of Neurociencies, University of Barcelona, 08028 Barcelona, Spain

**Keywords:** propionic acid, gut microbiota, metabolomics, cognitive decline, gut–brain axis, Alzheimer’s disease, dementia

## Abstract

The gut microbiome is involved in nutrient metabolism and produces metabolites that, via the gut–brain axis, signal to the brain and influence cognition. Human studies have so far had limited success in identifying early metabolic alterations linked to cognitive aging, likely due to limitations in metabolite coverage or follow-ups. Older persons from the Three-City population-based cohort who had not been diagnosed with dementia at the time of blood sampling were included, and repeated measures of cognition over 12 subsequent years were collected. Using a targeted metabolomics platform, we identified 72 circulating gut-derived metabolites in a case–control study on cognitive decline, nested within the cohort (discovery *n* = 418; validation *n* = 420). Higher serum levels of propionic acid, a short-chain fatty acid, were associated with increased odds of cognitive decline (OR for 1 SD = 1.40 (95% CI 1.11, 1.75) for discovery and 1.26 (1.02, 1.55) for validation). Additional analyses suggested mediation by hypercholesterolemia and diabetes. Propionic acid strongly correlated with blood glucose (r = 0.79) and with intakes of meat and cheese (r > 0.15), but not fiber (r = 0.04), suggesting a minor role of prebiotic foods per se, but a possible link to processed foods, in which propionic acid is a common preservative. The adverse impact of propionic acid on metabolism and cognition deserves further investigation.

## 1. Introduction

The gut microbiota, composed of micro-organisms (bacteria, archaea, bacteriophages, eukaryotic virus and fungi) that colonize the gut, plays an important role in human physiology and health [1,2]. The contribution of the microbial community to food digestion and nutrient metabolism is central to the biological functions of the host-microbiota system. Encoding specialized enzymes (e.g., for the breakdown of complex polysaccharides, polyphenols and synthesis of vitamins) that are not present in the human genome, the microbiome releases specific microbial and diet-derived metabolites in the gut and the bloodstream, such as pro-inflammatory factors, short-chain fatty acids, indole and phenolic metabolites and neurotransmitters [3]. 

There is a strong biological interplay between diet, the gut microbiota and the central nervous system, referred to as the gut–brain axis [4], which involves both direct gut-to-brain communication (through the nervus vagus), as well as more distant signaling via circulating metabolites in the systemic milieu; some of which cross the blood–brain barrier and exert direct neurobiological effects [5,6]. Emerging evidence even suggests a link between specific food- and gut microbiota-derived metabolites and cognitive aging and associated diseases, in particular dementia and its most frequent form, Alzheimer’s disease (AD) [7,8]. Candidate metabolites include anthranilic acid, a derivative of tryptophan produced by the kynurenic pathway [9,10]; trimethylamine oxide (TMAO), synthetized by gut microbiota from dietary betaine, choline and carnitine [11]; and cholesterol-derived biliary acids [12]. Short-chain fatty acids (SCFA), produced by microbial fermentation of certain dietary fibers, are also key metabolites of the gut–brain axis, which have been implicated in AD, e.g., by interfering with the protein–protein interactions necessary for toxic amyloid beta (Aβ) and aggregate formation [13]. They have also been involved in neuroinflammation, a critical underlying pathway of the gut–brain axis [14]. 

However, evidence of the associations between microbial metabolites and cognitive ageing in humans has so far been fragmented. Detection of diet- and gut-derived metabolites deserves highly sensitive metabolomics approaches, which have been developed only recently and have not yet been applied on a large scale in biomedical research [15]. Furthermore, studies have so far been limited in sample size and in the number of candidate metabolites analyzed, often without a replication stage. Moreover, causality is a critical issue in microbiome research [16]. Given that studies have so far been cross-sectional, they cannot infer whether diet- or microbiota-related metabolic changes are causally related to brain diseases or are a consequence. 

Therefore, identifying blood biomarkers before the onset of cognitive decline in long-term prospective studies is an essential step to identify early gut-derived metabolic alterations that potentially lead to cognitive aging and dementia. It is important to replicate discovery findings in populations with different ages and geographic origins, who have different environmental exposures and lifestyle, to ensure the robustness and external validity of results [17]. 

By using a case–control study nested in a large population-based cohort of older persons, we investigated the continuum of changes related to brain aging on a global scale (which results from both normal aging and brain pathologies, such as AD), focusing on long-term cognitive decline. We applied a large-scale and quantitative multi-metabolite platform to serum samples to determine circulating levels of 72 food- and gut microbiota-derived metabolites and studied their association with cognitive decline. 

## 2. Materials and Methods

### 2.1. Study Population

The Three-City (3C) study is a French cohort initiated in 1999 with the primary aim of studying vascular risk factors for dementia. It included 9294 non-institutionalized community dwellers aged 65 years or over from the following three French cities: Bordeaux (*n* = 2104), Dijon (*n* = 4931) and Montpellier (*n* = 2259) [18]. The 3C protocol was approved by the Consultative Committee for the protection of persons participating in biomedical research at Kremlin-Bicêtre University hospital in France and all participants provided written informed consent. At baseline, face-to-face interviews were conducted to collect socio-demographic data, lifestyle and health parameters. In addition, anthropometric and blood pressure measurements were performed, as well as fasting blood sampling for the constitution of a biobank. The number of medications regularly consumed by participants was recorded. Follow-up visits were conducted at home every two to three years. At baseline and at each follow-up visit, a battery of cognitive tests was performed by a certified and experienced neuropsychologist [18]. 

In Bordeaux, a nutritional survey was performed by a trained dietitian during a home interview conducted at the first follow-up in 2001–2002, including a food frequency questionnaire and a 24 h recall [19,20].

### 2.2. Nested Case–Control Samples

We worked with two different study centers (in Bordeaux and Dijon cities, France) to define separate samples (for discovery and validation stages, respectively) of a nested case–control study on serum metabolome and cognitive decline. Eligible participants showed no signs of dementia at baseline, had available serum samples in the biobank and had undergone at least one repeated cognitive evaluation over the subsequent twelve years of follow-up [21]. The averages of Z-scores of five neuropsychological tests (Mini-Mental State Examination [22], Benton Visual Retention Test [23], Isaacs’s Set Test [24], Trail-Making Test part A [25] and Trail-Making Test part B [25]) were used to define a composite score of global cognition. Individual slopes of cognitive change in the cognitive composite were computed using linear mixed models. Cases were defined as the participants with the worst slopes of cognitive change, and each case was matched to a control (i.e., a participant with a slope of cognitive change better than the population median) based on age (±3 years), sex and educational level (lower or higher than secondary school). In total, 209 cases in Bordeaux were successfully matched to a control, leading to a discovery sample of 418 participants. Similarly, in Dijon, 212 cases were successfully matched to a control, amongst whom 2 had no metabolomics measurement available and were excluded, leading to a validation sample of 420 participants.

### 2.3. Metabolomics Analysis of Serum Samples

Targeted metabolomics was conducted using a quantitative multi-metabolite platform for the simultaneous detection and quantification of 206 food-related metabolites, gut microbiota derivatives and endogenous metabolites (Supplementary Table S1) [26]. 

Serum samples were first subjected to protein precipitation with methanol containing 0.1% formic acid, followed by acetonitrile extraction, as previously described [21]. Supernatants were transferred to 96-well injection plates after addition of a set of internal standards. Analyses were carried out by ultra-high performance liquid chromatography coupled to tandem mass spectrometry (UHPLC-MS/MS), using the operating conditions described elsewhere [26]. Calibration curves were prepared at 10 concentration levels in the range 0.01–1000 µg/L.

The concentration of metabolites known to be influenced by pre-analytical factors (e.g., improper handling/storage of blood samples) was checked for the absence of abnormal values (±3 × IQR). The coefficients of variation for peak areas, retention times and peak widths of the internal standards were computed to evaluate the reproducibility. We assumed missing values to be mostly due to concentrations under the limit of quantification. To reduce the influence of missing values in the analysis, we studied only metabolites with ≥50% non-missing values in either the cases or the controls. The remaining missing values were imputed to zero.

In this study, we focused on 72 out of the 206 metabolites of the targeted metabolomics platform that are known to be produced or influenced by the gut microbiota, including aromatic amino acids and derivatives, biogenic quaternary amines, secondary bile acids, B-group vitamins, SCFA and metabolites derived from the gut biotransformation of dietary polyphenols (Supplementary Table S2).

### 2.4. Other Variables

Covariates were considered at the time of blood drawn at the baseline. They included smoking (in pack-years), alcohol consumption (in grams per day), body mass index (BMI, computed as weight/height^2^ (kg/m)), hypercholesterolemia (fasting blood cholesterol ≥ 6.2 mmol/L and/or taking lipid-lowering medication), diabetes (defined as fasting glycemia ≥ 7 mmol/L and/or taking diabetes medication) and hypertension (blood pressure ≥ 140/90 and/or antihypertensive treatment). 

### 2.5. Statistical Analyses

In the primary analysis dedicated to metabolite discovery, associations between the concentration (normalized) of each metabolite and the odds of cognitive decline were investigated using logistic regressions conditioned for matching variables (defined as the strongest risk factors for cognitive decline in older persons, i.e., age, sex and educational level), with correction for multiple testing based on the Benjamini–Hochberg false discovery rate (FDR) [27]. Since the use of an independent validation stage lowers the risk for false positives, we allowed for the discovery stage a permissive FDR-adjusted threshold of FDR < 0.15 for selection in subsequent validation. Selected metabolites (with *p* < 0.15 in the discovery stage) were tested for replication, using conditional logistic regressions. The statistical threshold for the validation stage was set at α = 0.05. In all logistic regression models, the log-linearity hypothesis was tested, and the metabolites that were not log-linearly associated with the odds of cognitive decline were transformed by the highest performing polynomials [28].

A secondary set of analyses was conducted on the metabolites identified and validated in the primary stage, considering lifestyle and cardio-metabolic conditions that could confound and/or mediate the relation of metabolites to cognitive decline. These secondary analyses were run using the pooled dataset (combining discovery and validation samples). First, a directed acyclic graph (DAG) was drawn; DAGs are visual representations of causal assumptions in epidemiological studies that help determine which adjustment variables should be included in the models. DAG methodology requires the *a priori* establishment of the potential confounders (i.e., variables that are associated with both the exposure(s) and the outcome but are not on the same causal path) and mediators (i.e., variables that are on the causal path between exposure(s) and outcome) of the relation under study. The list of potential confounders and mediators and the DAG were determined based on existing knowledge. Next, a multivariable-adjusted analysis was performed, including adjustment factors as determined by the DAG. Then, formal causal mediation analysis was carried out using counterfactual mediation analysis for matched data, to estimate the natural direct effect (NDE) of the selected metabolites on cognitive decline and their natural indirect effect (NIE) through potential mediators. We adapted the method proposed by Kim et al. [29,30] to several mediators modeled non-concomitantly in conditional logistic regression models (one for each mediator), while adjusting for both potential confounders and other mediators. 

Missing data for covariates (≤5% for all variables, in both samples) were imputed by multiple imputations (using chained equations with the fully conditional specification method; M = 5 imputations), whenever necessary. 

Statistical analyses were performed using SAS version 9.4 (SAS Institute Inc., Cary, NC, USA), R version 4.0.3 (R Foundation, Vienna, Austria) and DAGITTY version 3.0 for DAG construction.

## 3. Results

Cases and controls from both the discovery and validation samples were approximately 76 years old, on average, with 66% and 63% female participants, respectively, and 71% with an education level above secondary school in both samples (Table 1). Baseline characteristics were generally comparable between the two samples, except for hypertension, which was slightly more prevalent in the validation sample (Dijon). In both samples, diabetes was significantly higher in cases than in controls (13% versus 6%, *p* = 0.01), while the other characteristics did not significantly differ by case–control status (*p* > 0.05). 

In the discovery stage, seven food- and gut-microbiota-derived metabolites associated with the odds of cognitive decline were selected (Table 2), including three amino acid derivatives (phenylacetylglutamine, indolelactic acid and kynurenic acid); a TMAO substrate (betaine), a vitamin B (pantothenic acid), a SCFA (propionic acid) and a polyphenol derivative (3′,4′-DHPV-S). Among these, only propionic acid was replicated in the validation stage (see distribution in Supplementary Figure S1). For each 1 standard deviation (SD) increase in propionic acid concentration in serum, the odds of cognitive decline increased by 40% in the discovery sample (OR = 1.40, 95% CI 1.11–1.75, FDR-corrected *p* = 0.07) and by 26% in the validation sample (OR = 1.26, 95% CI 1.02–1.55, *p* = 0.03) (Table 2). 

**Figure 1:**
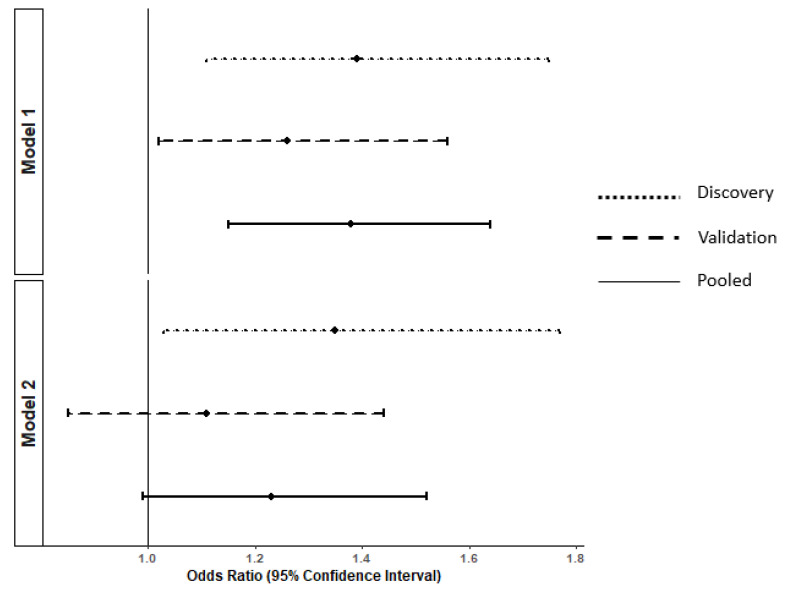
Multivariable-adjusted associations ^1^ between standardized concentration of propionic acid in serum and the odds of subsequent cognitive decline over 12 years in the discovery (*n* = 418), validation (*n* = 420) and pooled samples (*n* = 838). ^1^ Estimated using conditional logistic regressions (conditioned using matching variables, i.e., age, sex and educational level). Model 1: adjusted for potential confounders (alcohol consumption and smoking). Model 2: adjusted for confounders (alcohol consumption and smoking) and potential mediators (body mass index, hypertension, hypercholesterolemia, and diabetes).

For multivariable-adjusted analyses, we considered lifestyle factors (smoking and alcohol consumption) and cardio-metabolic risk factors (BMI, hypertension, hypercholesterolemia and diabetes) as covariates of interest. First, we tested interactions of propionic acid with each of these factors regarding cognitive decline and all were found to be non-statistically significant (*p* > 0.15). Second, as there was suggestive evidence in the literature that cardio-metabolic health could mediate the relation of propionic acid to cognitive decline [31,32,33,34], we established a DAG with alcohol consumption and smoking as potential confounders and BMI, hypertension, hypercholesterolemia and diabetes as potential mediators (Supplementary Figure S2). After adjustment for potential confounders (Figure 1, model 1), propionic acid remained significantly associated with cognitive decline (OR = 1.38, 95% CI 1.15–1.64 in the pooled sample). When adjusting subsequently for potential mediators (Figure 1, model 2), the association was attenuated and did not reach significance in the pooled sample (OR = 1.23, 95%CI 0.99–1.52). Adjusting for the number of regularly consumed medications did not modify the results.

When we further deconstructed the effect of propionic acid on cognitive decline with mediation analyses (Supplementary Figure S3), the direct effect (NDE) of propionic acid (coded as >75th versus <25th percentiles) on the odds of cognitive decline was 1.75 (95% CI 1.01, 3.09). The indirect effect (NIE) operating through hypercholesterolemia was statistically significant (1.33 95% CI (1.05, 1.94)) and the one operating through diabetes was borderline non-significant (1.17 (0.99, 1.52)). The percentage of mediation (NIE/total effect) from hypercholesterolemia was 34% and from diabetes, it was 21%. The mediating effects of BMI (defined as ≥ versus < 30 kg/m^2^) and of hypertension were not statistically significant (OR for NIE through BMI = 1.06 (0.87, 1.36) and OR for NIE through hypertension = 1.69 (0.92, 3.47)). 

Fasting blood glucose levels were strongly correlated with the concentration of propionic acid (Pearson’s correlation = 0.79, *p* < 0.001 in the pooled sample) and when using blood glucose instead of diabetes in mediation analyses (defined as ≥ versus < median value (4.9 mmol/L)), mediation by glycemia was statistically significant (OR for NDE of high versus low propionic acid = 2.26 (1.36, 4.32) and OR for NIE through blood glucose = 2.03 (1.24, 3.86)).

## 4. Discussion

We adopted a novel epidemiological approach to the gut–brain axis through evaluation of a large panel of circulating post-biotics in relation to subsequent long-term cognitive decline, in a population-based cohort. Among the 72 metabolites determined using a multi-metabolite UHPLC-MS/MS analysis, 7 were associated with the odds of cognitive decline in the discovery stage, including 3 amino acid derivatives (phenylacetylglutamine, indolelactic acid and kynurenic acid); a TMAO substrate (betaine), a B vitamin (pantothenic acid), a SCFA (propionic acid) and a polyphenol derivative (3′,4′-DHPV-S). Among these, only propionic acid was significantly associated with cognitive decline in the validation stage. Finding a consistent association between propionic acid and cognitive decline in two samples of different geographic origin (south-west and north-east of France for discovery and validation, respectively), exposed to different environments, neutralizes some potentially unmeasured confounders, and supports the robustness of our results. Overall, for each increase of 1-SD of propionic acid in serum (i.e., approximately 20 µg/L), the odds of cognitive decline increased by 26% and 40% in the validation and discovery samples, respectively. Additionally, in secondary exploratory mediation analyses, we found a potentially mediating role of cardiometabolic conditions, specifically hypercholesterolemia and fasting glycemia (diabetes with borderline significance). 

Propionic acid is a SCFA produced by *Bacteroidetes* and *Firmicutes* spp. via colonic fermentation of dietary fibers and undigested polysaccharides, which are found in high concentrations in prebiotic foods (e.g., artichokes, leeks, salsify, onions and apples) [35]. It is estimated that in a human being who weighs 85 kg, the gut microbiota produces approximately 29.5 mg/kg/day of propionate via colonic fermentation [36]. In addition to gut fermentation, the two other sources of propionate are the oral microbiome and the intake of processed, packaged foods, such as bread and cheese, where it is incorporated as a preservative (i.e., anti-microbial agent). Hence, in the US, propionic acid has been found in concentrations of 0.1% to 0.4% in foods such as baked goods, dairy and meat products, puddings, gelatins and jams [31,37,38], meaning that most people are exposed to dietary sources of propionate daily. In our study, serum propionic acid was not correlated with intake of dietary fiber (r = 0.04, *p* = 0.41 in Bordeaux sample), but was significantly correlated with meat and cheese intake (Pearson’s correlation = 0.18 and 0.15; *p* = 0.001 and 0.005, respectively). This may indirectly suggest that the exogenous origin of food preservatives may contribute to circulating propionic acid in our population. Regardless of the source (either synthesized by the microbiota or of exogenous origin from processed food), most of the propionate produced/ingested reaches blood circulation [39]; 90% is metabolized by the liver [40,41] and part of the remaining fraction crosses the blood–brain barrier through the GRP41 transporter [42]. However, human data on SCFA bioavailability, including propionic acid, are very limited due to the volatile nature of these metabolites [43,44].

Propionic acid exerts many physiological functions, including its role in the promotion of enteric smooth muscle contractions and in immune function [45,46]. It also plays a role in glucose and energy metabolism, serving as a substrate for gluconeogenesis (via the succinate pathway) [31,47]. However, the effect of propionic acid on brain health and aging is still poorly understood. Our results provide novel findings that suggest an adverse impact of propionic acid on cognitive aging, in a controversial area. On the one hand, our findings are in contradiction with studies that have found that SCFAs, including propionate, benefit both the integrity of the blood–brain barrier via the GRP41 receptor [13,48] and the maturation and function of brain microglia. SCFAs also exert anti-inflammatory activity, especially in acute neurological conditions (e.g., ischemic stroke) [49]. They decrease pro-inflammatory cytokines both in vitro and in mice studies [14]. The role of SCFAs in neurodegenerative diseases has been controversial, with, for example, some studies suggesting that SCFAs may decrease [44,50,51] and others suggesting that they may promote [52,53] Aβ aggregation (the primary AD neuropathology), potentially via activation of microglial activity. Our findings are consistent with two small studies that reported increased propionic acid concentrations in saliva and cerebrospinal fluid of AD patients [54,55]. A study also reported a greater amount of *Bacteroides* (the bacteria phylum that produces propionic acid) in the microbiota of AD patients compared to healthy subjects [56]. 

A novel concept raised by our study is the mediation of the adverse association of propionic acid to cognitive decline by blood glucose, suggesting metabolic disruption as a central pathway in the relation of propionic acid to cognitive aging. Impaired metabolic health (i.e., hyperglycemia and insulin resistance) is an important risk factor for cognitive decline and dementia [57], and in our population, serum propionic acid was strongly correlated with blood glucose. This is consistent with recent preclinical and clinical studies where oral administration of propionic acid had potent hyperglycemic effects (i.e., increased glucose production, insulin secretion and glycogenolysis). For example, in a small randomized controlled trial of 28 healthy volunteers, consuming propionic acid (1500 mg calcium propionate) under various metabolic conditions led to metabolic alterations, suggesting inappropriate activation of the insulin counter-regulatory hormonal network [58]. In addition, although recognized as safe by the US food and drug administration [59], there is recent evidence that exogenous propionic acid (when taken orally) may be a metabolic disruptor [58]. The dietary sources of propionic acid used as a preservative (processed meats, dairy products and sweets) are also components of the Western diet, which is a risk factor of dementia and cognitive decline [60]. It is, thus, possible that the association found with propionic acid in our study is a surrogate marker of a more general deleterious impact of Western diets on cognitive health. The mediation found not only with diabetes, but also with hypercholesterolemia in our study would favor this hypothesis, as Western diets were reported to induce dyslipidemia and insulin resistance [61,62], which in turn may increase odds of developing cognitive decline. 

There are other potential metabolic pathways that link propionic acid to brain health that may deserve further exploration. Of these are ammonia and glutamate, two metabolites with inter-related cerebral metabolism [63], which are both (i) increased in the brain in response to higher propionic acid [43,64] and (ii) altered in AD pathology [65,66]. Hence, higher brain ammonium levels have been reported in AD patients, while glutamate, a major excitatory neurotransmitter, has an established excitotoxicity in AD [65,66]. Thus, propionic acid may have a neurotoxic effect, increasing both ammonium-related cerebral energetic defects and glutamate excitotoxicity. Another metabolite of interest in the pathway from propionic acid to cognitive health is kynurenic acid. It is endogenously produced from tryptophan and inhibits the excessive release of glutamate during excitotoxicity [67]; thus, the protective association of kynurenic acid with cognitive health is expected. Nevertheless, in our discovery stage, kynurenic acid was associated with higher cognitive decline, potentially reflecting a compensatory mechanism against increased glutamate neurotoxicity, although the findings were not replicated. In addition, caution in the interpretation of our correlational findings in terms of central pathways is warranted, as for some of these metabolites, blood measures poorly reflect intracerebral concentrations (e.g., blood glutamate is 100 to 200 times less concentrated in the blood than brain [68]). Therefore, using these blood biomarkers, we were not able to accurately evaluate mediation by these central pathways and we limited our investigation of potential mediation to peripheral metabolism. 

Our study has important strengths, including the use of two population-based samples from independent study sites for both discovery and validation; an assessment of a large panel of circulating post-biotics, using a unique multi-metabolite platform with sensitive, cutting-edge metabolomics technology (UHPLC-MS/MS); and a prospective evaluation of cognitive decline over more than a decade. This enabled a broad overview of the metabolic pathways that underlie the gut–brain axis. Moreover, the prospective design ensured that any of the biological changes observed in participants that did not exhibit signs of dementia at the time of blood sampling preceded cognitive deterioration, and thus allowed the identification of early gut–brain axis markers of cognitive aging, while minimizing the risk of reverse causality (which may occur when cognitive changes influence behaviors, such as diet, and related biology, including gut microbiota activity). 

Despite many strengths, this study also has some limitations. First, while we tried to minimize the risk of false positives through the use of an external validation stage, the risk of false negatives could not be dismissed completely. Indeed, the sample size was relatively high for a UHPLC-MS/MS study, but could still be insufficient to ensure adequate statistical power to detect differences in the levels of metabolites with low circulating basal values and high inter- and intra-individual variability. Moreover, serum samples were analyzed at the study baseline only; therefore, we could not examine longitudinal changes in the serum metabolome during the course of cognitive decline. Finally, although the metabolite panel was large, it did not cover all metabolites of the gut–brain axis (due to the lack of available standards, or detection limitations by UHPLC-MS/MS technologies). For example, some microbiota-derived metabolites that are potentially related to cognition, such as γ-aminobutyric acid (GABA), ammonium, some phytoestrogens and oligosaccharides, such as lipopolysaccharide (LPS), were not quantified.

## 5. Conclusions

In conclusion, in this novel exploration of the gut–brain axis, we measured a large panel of post-biotics in a cohort of older persons and found an association between increased circulating propionic acid levels and higher cognitive decline. Propionic acid may be derived from the fermentation of undigested dietary fiber by the gut microbiota, as well as from dietary intake, as it is a common food preservative in processed food such as meat, dairy products and sweets. Moreover, we found preliminary evidence to support the potential mediation by impaired cardio-metabolic health (diabetes and hypercholesterolemia), which deserves further research.

## Figures and Tables

**Table 1:** Baseline characteristics of cases of cognitive decline over 12 years and matched controls in the discovery and validation samples.

	Discovery (*n* = 418)		Validation (*n* = 420)	
	Cases	Controls	Cases	Controls
Matching variables				
Age (years), mean (SD)	75.9 (4.4)	75.7 (4.2)	76.5 (5.2)	76.1 (4.7)
Women	138 (66)	138 (66)	133 (63)	133 (63)
Level of education above secondary level	149 (71)	149 (71)	150 (71)	150 (71)
Baseline characteristics				
BMI (kg/m^2^), mean (SD)	26.8 (4.3)	26.1 (3.6)	25.7 (4.5)	25.0 (3.6)
Alcohol consumption (g per day), mean (SD)	13.0 (14.6)	14.6 (17.2)	12.3 (14.5)	12.4 (12.7)
Smoking (pack-years), mean (SD)	9.1 (19.7)	7.3 (14.6)	8.1 (19.2)	6.0 (12.8)
High blood pressure	164 (78)	159 (76)	176 (84)	174 (83)
Hypercholesterolemia	79 (38)	93 (44)	85 (40)	80 (38)
Diabetes	27 (13) *	12 (6) *	27 (13) *	12 (6) *
Number of medications, mean (SD)	4.9 (2.7) *	4.1 (2.4) *	5.5 (3.0) *	4.0 (2.9) *

Values represent sample sizes (percentage), unless otherwise stated. * Statistically significant difference (*p* < 0.05) between cases and controls, based on paired t-test for quantitative variables and on McNemar test for matched data for qualitative variables.

**Table 2:** Associations ^1^ between standardized concentrations of food- and gut microbiota-derived metabolites in serum and the odds of subsequent cognitive decline over 12 years in discovery and validation stages.

	Discovery (*n* = 418)			Validation (*n* = 420)		
Metabolite	OR ^2^	95% CI	FDR-Adjusted *p* Value ^3^	OR	95% CI	*p* Value
Phenylalanine	0.93	0.76; 1.14	0.83			
Tyrosine	1.18	0.97; 1.44	0.37			
Tryptophan	1.11	0.92; 1.35	0.70			
Phenyl-lactic acid	1.26	1.02; 1.57	0.26			
*p*-HPLA	-	-	0.33			
**Phenylacetylglutamine**	1.34	1.08; 1.66	0.09 *	1.14	0.94; 1.39	0.19
Epinephrine	-	-	0.84			
*p*-Cresol-G	1.13	0.93; 1.36	0.64			
*p*-Cresol-S	1.13	0.93; 1.37	0.68			
Indoxyl-S	-	-	0.80			
Serotonin	0.96	0.79; 1.15	0.87			
**Indolelactic acid**	1.38	1.11; 1.72	0.07*	0.93	0.77; 1.12	0.46
Indoleacetic acid	1.12	0.91; 1.37	0.70			
5-HIAA	-	-	0.84			
Indolepropionic acid	1.00	0.84; 1.20	1.00			
Kynurenine	1.17	0.95; 1.43	0.49			
**Kynurenic acid**	1.34	1.07; 1.67	0.10 *	1.07	0.88; 1.29	0.49
Xanthurenic acid	1.12	0.92; 1.37	0.68			
Anthranilic acid	0.97	0.80; 1.18	0.94			
Picolinic acid	0.93	0.77; 1.12	0.80			
Ergothioneine	0.98	0.81; 1.18	0.94			
Lactic acid	1.10	0.91; 1.34	0.76			
Choline	1.19	0.97; 1.45	0.37			
TMAO	1.03	0.84; 1.26	0.94			
**Betaine**	0.73	0.60; 0.88	0.04 *	0.97	0.8; 1.17	0.73
Carnitine	1.07	0.88; 1.31	0.83			
GDCA	1.24	1.01; 1.52	0.26			
Thiamine	-	-	0.92			
Riboflavin	0.87	0.71; 1.07	0.59			
Niacinamide	1.26	1.03; 1.55	0.21			
**Pantothenic acid**	1.43	1.15; 1.77	0.04 *	1.04	0.86; 1.24	0.70
4-pyridoxic acid	-	-	0.94			
Biotin	1.38	1.03; 1.86	0.26			
**Propionic acid**	1.40	1.11; 1.75	0.07 *	1.26	1.02; 1.55	0.03
Butyric acid	1.08	0.90; 1.30	0.80			
Valeric acid	0.93	0.76; 1.13	0.80			
2-HBA	1.10	0.87; 1.38	0.80			
3-HBA-S	0.99	0.82; 1.19	0.99			
4-HBA-S	1.06	0.87; 1.29	0.84			
2,6-DHBA	1.06	0.88; 1.29	0.84			
3,4-DHBA	0.95	0.77; 1.16	0.87			
HA	0.82	0.67; 1.02	0.33			
4-HHA	0.95	0.78; 1.16	0.87			
3-HHA	0.98	0.79; 1.20	0.94			
iVA	1.01	0.83; 1.22	0.99			
2-HPAA	1.08	0.89; 1.31	0.80			
4-HPAA-G	1.18	0.94; 1.47	0.50			
3-HPAA-S	1.00	0.82; 1.20	0.99			
3,4-DHPAA-S	1.00	0.82; 1.21	0.99			
FA-S	0.95	0.78; 1.16	0.87			
3-HPPA	0.96	0.78; 1.17	0.89			
HPPA-S	0.91	0.74; 1.13	0.80			
3,5-DHPPA-S	1.02	0.85; 1.23	0.94			
DHCA-3S	1.03	0.84; 1.25	0.94			
DHFA	1.11	0.91; 1.35	0.76			
DHFA-S	0.89	0.73; 1.08	0.68			
DHiFA-S	1.00	0.83; 1.22	0.99			
3-HPHPA	1.01	0.83; 1.23	0.99			
PYR-S	0.99	0.82; 1.20	0.99			
MePYR-S	0.97	0.80; 1.18	0.94			
CAT-S	-	-	0.80			
4-MeCAT-S	0.84	0.66; 1.05	0.47			
VAN	0.82	0.66; 1.02	0.33			
**3′,4′-DHPV-S**	0.70	0.54; 0.91	0.09 *	1.04	0.86; 1.25	0.69
MHPV-S	0.83	0.68; 1.02	0.33			
UroA-G	0.92	0.75; 1.14	0.80			
UroA-S	0.89	0.71; 1.11	0.71			
UroB-G	1.04	0.86; 1.27	0.89			
UroB-S	1.05	0.86; 1.28	0.87			
DHRSV-S	0.98	0.80; 1.19	0.94			
EL	0.87	0.71; 1.06	0.51			
EL-S	0.83	0.69; 1.01	0.33			

^1^ Estimated using conditional logistic regressions (conditioned using matching variables, i.e., age, sex and educational level). ^2^ For 1 SD increase in metabolite concentration, when used continuously (standardized). Note that in the case of non-log-linear relationships between a metabolite and the odds of cognitive decline, a transformation into fractional polynomials was used to satisfy the model assumptions. The association of the metabolite with the odds of cognitive decline was then estimated with a combination of model parameters. Since results cannot be interpreted based on single fractional polynomial parameters but on the overall polynomial function, for the sake of simplicity, these parameters were not provided (OR missing in table). The *p*-value associated with the log-likelihood ratio test of all parameters of the polynomial function together (i.e., testing the global effect of the metabolite on the odds of cognitive decline) is presented. ^3^ *p*-value after Benjamini–Hochberg false discovery rate correction. Metabolites with *p*-values ≤ 0.15 highlighted with a asterisk in the discovery stage were selected for the validation stage. Abbreviations: 2,6-DHBA: 2,6-dihydroxybenzoic acid; 2-HBA: 2-hydroxybenzoic acid, 2-HPAA: 2-hydroxyphenylacetic acid, 3,4-DHBA: 3,4-dihydroxybenzoic acid; 3,4-DHPAA-S: 3,4-dihydroxyphenylacetic acid sulfate; 3,5-DHPPA-S: 3-(3,5-dihydroxyphenyl)propionic acid sulfate; 3′,4′-DHPV-S: 3′,4′-dihydroxyphenyl-γ-valerolactone sulfate; 3-HBA-S: 3-hydroxybenzoic acid sulfate; 3-HHA: 3-hydroxyhippuric acid; 3-HPAA-S: 3-hydroxyphenylacetic acid sulfate; 3-HPHPA: 3-(3-hydroxyphenyl)-3-hydroxypropionic acid; 3-HPPA: 3-(3-hydroxyphenyl)propionic acid; 4-HBA-S: 4-hydroxybenzoic acid sulfate; 4-HHA: 4-hydroxyhippuric acid; 4-HPAA-G: 4-hydroxyphenylacetic acid glucuronide; 4-MeCAT-S: 4-methylcatechol sulfate; 5-HIAA: 5-hydroxyindole-3-acetic acid; CAT-S: catechol sulfate; CI: confidence interval; DHCA-3S: dihydrocaffeic acid 3-sulfate; DHFA: dihydroferulic acid; DHFA-S: dihydroferulic acid 4-sulfate; DHiFA-S: dihydroisoferulic acid 3-sulfate; DHRSV-S: dihydroresveratrol sulfate; EL: enterolactone; EL-S: enterolactone sulfate; FA-4S: ferulic acid 4-sulfate; GDCA: glycodeoxycholic acid; HA: hippuric acid; HPPA-S: hydroxyphenylpropionic acid sulfate; IAA: indole-3-acetic acid; IPA: indole-3-propionic acid; iVA: isovanillic acid; MePYR-S: methylpyrogallol sulfate; MHPV-S: 4′-hydroxy-3′-methoxyphenyl-γ-valerolactone sulfate; OR: odds ratio; p-cresol-G: p-cresol glucuronide; p-cresol-S: p-cresol sulfate; p-HPLA: p-hydroxyphenyl-lactic acid; PYR-S: pyrogallol sulfate; TMAO: trimethylamine N-oxide; UroA-G: urolithin A glucuronide; UroA-S: urolithin A sulfate; UroB-G: urolithin B glucuronide; UroB-S: urolithin B sulfate; VAN: vanillin.

## Data Availability

The data presented in this study are available upon request from the corresponding author.

## Supplementary Materials

The following supporting information can be downloaded at: https://www.mdpi.com/article/10.3390/nu14214688/s1, Figure S1: Distribution of plasmatic propionic acid concentration in the pooled sample (n = 838); Figure S2: Directed Acyclic Graph to identify potential confounders and mediators in the relation of propionic acid in serum to cognitive decline; Figure S3: Counterfactual mediation analysis for matched data to estimate the mediating effect of hypertension, hypercholesterolemia and diabetes in the relation of propionic acid in serum to the odds of cognitive decline over 12 years in the pooled sample (n = 838); Table S1: List of the 206 food-related metabolites quantified in serum by the multi-metabolite metabolomics platform (with the 72 candidate food and gut microbiota-derived metabolites emphasized in bold); Table S2: List of the 72 candidate food and gut microbiota-derived metabolites measured in serum, with chemical class, endogenous versus exogenous origin and relation to the gut microbiota (derivative versus substrate).
