# Supplementary material for: Exploration of the Gut–Brain Axis through Metabolomics Identifies Serum Propionic Acid Associated with Higher Cognitive Decline in Older Persons
Source: Nutrients. 2022 Nov 5;14(21):4688. doi: 10.3390/nu14214688 (PMC9655149; doi:10.3390/nu14214688)
Supplement: Supplementary file 1 [file nutrients-14-04688-s001.zip › nutrients-2004684-supplementary.pdf]

**Exploration of the gut-brain axis through metabolomics identifies serum propionic acid associated with higher cognitive decline in older persons**

Neuffer J. & al

Supplementary data

**Supplementary table S1.** List of the 206 food-related metabolites quantified in serum by the multi-metabolite metabolomics platform (with the 72 candidate food and gut microbiota-derived metabolites emphasized in bold)

| Endogenous metabolites |
|---|
| glycine |
| L-alanine |
| L-valine |
| L-leucine |
| L-serine |
| L-threonine |
| L-proline |
| L-arginine |
| L-glutamate |
| L-aspartate |
| L-glutamine |
| L-asparagine |
| L-histidine |
| L-lysine |
| L-cysteine |
| L-methionine |
| **L-phenylalanine** |
| **L-tyrosine** |
| **L-tryptophan** |
| pyroglutamic acid |
| N6-trimethyl-lysine |
| **phenyllactic acid** |
| **p-hydroxyphenyllactic acid** |
| **phenylacetylglutamine** |
| **epinephrine** |
| **p-cresol sulfate** |
| **p-cresol glucuronide** |
| **indoxyl sulfate** |
| 5-hydroxytryptophan |
| **serotonin** |
| **indole-3-lactic acid** |
| **indole-3-acetic acid** |
| **5-hydroxyindole-3-acetic acid** |

**indole-3-propionic acid**
**kynurenine**
**kynurenic acid**
**xanthurenic acid**
**anthranilic acid**
**picolinic acid**
oxaloacetic acid
citric acid
**lactic acid**
pyruvic acid
**propionic acid**
**butyric acid**
**valeric acid**
octanoic acid
decanoic acid
lauric acid
myristic acid
pentadecanoic acid
palmitic acid
palmitoleic acid
margaric acid
stearic acid
oleic acid
linoleic acid
linolenic acid
eicosapentaenoic acid
arachidonic acid
docosatetraenoic acid
docosapentaenoic acid
docosahexaenoic acid
D-glucose
ornithine
citrulline
**choline**
**trimethylamine N-oxide**
**betaine**
taurine
urea
creatine
creatinine
guanosine
xanthine
uric acid
allantoin
hypoxanthine
adenine

adenosine
uracil
uridine
**L-carnitine**
Acetyl-L-carnitine
Propionyl-L-carnitine
Butyryl-L-carnitine
Hexanoyl-L-carnitine
Octanoyl-L-carnitine
Decanoyl-L-carnitine
Undecanoyl-L-carnitine
Dodecanoyl-L-carnitine
Myristoyl-L-carnitine
Palmitoyl-L-carnitine
Oleoyl-L-carnitine
Linoleyl-L-carnitine
Desmosterol
7-Ketocholesterol
Cholesterol sulfate
pregnenolone sulfate
dehydroepiandrosterone sulfate
testosterone
estrone 3-sulfate
17b-estradiol 3-sulfate
cortisol
corticosterone
11-deoxycortisol
**Cholic acid**
Glycocholic acid
Taurocholic acid
Chenodeoxycholic acid
Chenodeoxycholic acid 3-glucuronide
Glycochenodeoxycholic acid
Glycochenodeoxycholic acid 3-glucuronide
Taurochenodeoxycholic acid
**Deoxycholic acid**
**Glycodeoxycholic acid**
Glycodeoxycholic acid 3-sulfate
**Taurodeoxycholic acid**
**Glycolitocholic acid 3-sulfate**
**Taurolitocholic acid 3-sulfate**
Retinol
**Thiamine**
**Riboflavin**
**Niacinamide**
**pantothenic acid**

| |
|---|
| **4-Pyridoxic acid** |
| **Biotin** |
| ascorbic acid |
| 25-hydroxyvitamin D3 |
| α-Tocopherol |
| lynoleoyl-glycerophosphocholine |
| **exogenous metabolites** |
| **2-hydroxybenzoic acid** |
| **3-hydroxybenzoic acid sulfate** |
| **4-hydroxybenzoic acid sulfate** |
| **2,6-dihydroxybenzoic acid** |
| **3,4-dihydroxybenzoic acid** |
| **dihydroxybenzoic acid glucuronide** |
| **hippuric acid** |
| **4-hydroxyhippuric acid** |
| **3-hydroxyhippuric acid** |
| **vanillic acid** |
| **isovanillic acid** |
| **2-hydroxyphenylacetic acid** |
| **4-hydroxyphenylacetic acid glucuronide** |
| **3-hydroxyphenylacetic acid sulfate** |
| **3,4-dihydroxyphenylacetic acid sulfate** |
| **o-coumaric acid sulfate** |
| **ferulic acid 4-sulfate** |
| **3-(3-hydroxyphenyl)propionic acid** |
| **hydroxyphenylpropionic acid sulfate** |
| **3-(3,5-dihydroxyphenyl)propionic acid sulfate** |
| **dihydrocaffeic acid 3-sulfate** |
| **dihydroferulic acid** |
| **dihydroferulic acid 4-sulfate** |
| **dihydroisoferulic acid 3-sulfate** |
| **3-(3-hydroxyphenyl)-3-hydroxypropionic acid** |
| **pyrogallol sulfate 1** |
| **methylpyrogallol sulfate 1** |
| **catechol sulfate** |
| **4-methylcatechol sulfate 1** |
| **vanillin** |
| **5-(4-hydroxy(3-4-dihydroxyphenyl)-valeric acid sulfate 1** |
| **3’,4’-dihydroxyphenyl-γ-valerolactone sulfate** |
| **4’-hydroxy-3’-methoxyphenyl-γ-valerolactone sulfate** |
| naringenin 7-glucuronide |
| naringenin glucuronide |

hesperetin 3'-glucuronide
hesperetin sulfate
bergaptol glucuronide
bergaptol sulfate
umbelliferone sulfate
4-methylumbelliferone sulfate
daidzein 4'-sulfate
genistein 7-sulfate
**urolithin A glucuronide**
**urolithin A sulfate**
**urolithin B glucuronide**
**urolithin B sulfate**
trans-resveratrol 3-sulfate
cis-resveratrol 3-sulfate
**dihydroresveratrol sulfate 1**
**enterolactone**
**enterolactone sulfate**
atractyligenin glucuronide
acesulfame K
saccharin
ethyl sulfate
tartaric acid
2-furoylglycine
1-methylxanthine
3-methylxanthine
paraxanthine +theofylline
theobromine
caffeine
1-methyluric acid
1,7-dimethyluric acid
5-acetamido-6-formylamino-3-methyluracil
cyclo(L-leucyl-L-prolyl)
cyclo(L-prolyl-L-valyl)
**ergothioneine**
proline betaine
4-hydroxyproline betaine
carnosine
1-methylhistidine
3-methylhistidine
arsenobetaine
N-methylpyridinium
α-chaconine

**Supplementary table S2.** List of the 72 candidate food and gut microbiota-derived metabolites measured in serum, with chemical class, endogenous versus exogenous origin and relation to the gut microbiota (derivative versus substrate)

| Class | Metabolite | Abbreviated name | Derivative (D)/ Substrate (S) |
|---|---|---|---|
| Amino-acids and derivatives | Phenylalanine | Phenylalanine | S |
| | Tryptophan | Tryptophan | S & D |
| | Tyrosine | Tyrosine | S & D |
| | Phenyl-lactic acid | Phenyl-lactic acid | D |
| | p-Hydroxyphenyl-lactic acid | p-HPLA | D |
| | Phenylacetylglutamine | Phenylacetylglutamine | D |
| | p-Cresol sulfate | p-cresol-S | D |
| | p-Cresol glucuronide | p-cresol-G | D |
| | Indole-3-propionic acid | IPA | D |
| | Indole-3-acetic acid | IAA | D |
| | Indoxyl sulfate | Indoxyl Sulfate | D |
| | Indole-3-lactic acid | Indolelactic acid | D |
| | 5-Hydroxyindole-3-acetic acid | 5-HIAA | D |
| | Kynurenine | Kynurenine | D |
| | Kynurenic acid | Kynurenic acid | D |
| | Xanthurenic acid | Xanthurenic acid | D |
| | Anthranillic acid | Anthranillic acid | S |
| | Picolinic acid | Picolinic acid | D |
| | Ergothioneine | Ergothioneine | D |
| | Epinephrine | Epinephrine | D |
| | Serotonin | Serotonin | D |
| Organic acids | Lactic acid | lactic acid | D |
| TMAO and substrates | Trimethylamine N-oxide | TMAO | D |
| | Betaine | betaine | S |
| | Choline | choline | S |
| | L-Carnitine | L-carnitine | S |
| Bile acids | Glycodeoxycholic acid | GDCA | D |
| B vitamins | Thiamine | Thiamine | D |
| | Riboflavin | Riboflavin | S&D |
| | Niacinamide | Niacinamide | D |
| | Pantothenic acid | Pantothenic acid | S&D |
| | 4-Pyridoxic acid | 4-pyridoxic acid | D |
| | Biotin | Biotin | D |
| Short chain fatty acids | Valeric acid | valeric acid | D |
| | Butyric acid | butyric acid | D |
| | Propionic acid | propionic acid | D |
| Phenolic compounds and derivatives | Urolithin A glucuronide | UroA-G | D |
| | Urolithin A sulfate | UroA-S | D |
| | Urolithin B glucuronide | UroB-G | D |
| | Urolithin B sulfate | UroB-S | D |
| | 2-Hydroxybenzoic acid | 2-HBA | D |
| | 3-Hydroxybenzoic acid sulfate | 3-HBA-S | D |

| Class | Metabolite | Abbreviated name | Derivative (D)/ Substrate (S) |
|---|---|---|---|
| | 4-Hydroxybenzoic acid sulfate | 4-HBA-S | D |
| | 2,6-Dihydroxybenzoic acid | 2,6-DHBA | D |
| | 3,4-Dihydroxybenzoic acid | 3,4-DHBA | D |
| | Hippuric acid | HA | D |
| | 4-Hydroxyhippuric acid | 4-HHA | D |
| | 3-Hydroxyhippuric acid | 3-HHA | D |
| | Isovanillic acid | iVA | D |
| | 2-Hydroxyphenylacetic acid | 2-HPAA | D |
| | 4-Hydroxyphenylacetic acid glucuronide | 4-HPAA-G | D |
| | 3-Hydroxyphenylacetic acid sulfate | 3-HPAA-S | D |
| | 3,4-Dihydroxyphenylacetic acid sulfate | 3,4-DHPAA-S | D |
| | Ferulic acid 4-sulfate | FA-4S | D |
| | 3-(3-Hydroxyphenyl)propionic acid | 3-HPPA | D |
| | Hydroxyphenylpropionic acid sulfate | HPPA-S | D |
| | 3-(3,5-Dihydroxyphenyl)propionic acid sulfate | 3,5-DHPPA-S | D |
| | Dihydrocaffeic acid 3-sulfate | DHCA-3S | D |
| | Dihydroferulic acid | DHFA | D |
| | Dihydroferulic acid 4-sulfate | DHFA-S | D |
| | Dihydroisoferulic acid 3-sulfate | DHiFA-S | D |
| | 3-(3-Hydroxyphenyl)-3-hydroxypropionic acid | 3-HPHPA | D |
| | Pyrogallol sulfate | PYR-S | D |
| | Methylpyrogallol sulfate | MePYR-S | D |
| | Catechol sulfate | CAT-S | D |
| | 4-Methylcatechol sulfate | 4-MeCAT-S | D |
| | Vanillin | VAN | D |
| | 3',4'-Dihydroxyphenyl-γ-valerolactone sulfate | 3',4'-DHPV-S | D |
| | 4'-Hydroxy-3'-methoxyphenyl-γ-valerolactone sulfate | MHPV-S | D |
| | Dihydroresveratrol sulfate | DHRSV-S | D |
| | Enterolactone sulfate | EL-S | D |
| | Enterolactone | EL | D |

**Supplementary Figure S1**. Distribution of plasmatic propionic acid concentration in the pooled sample (n=838)

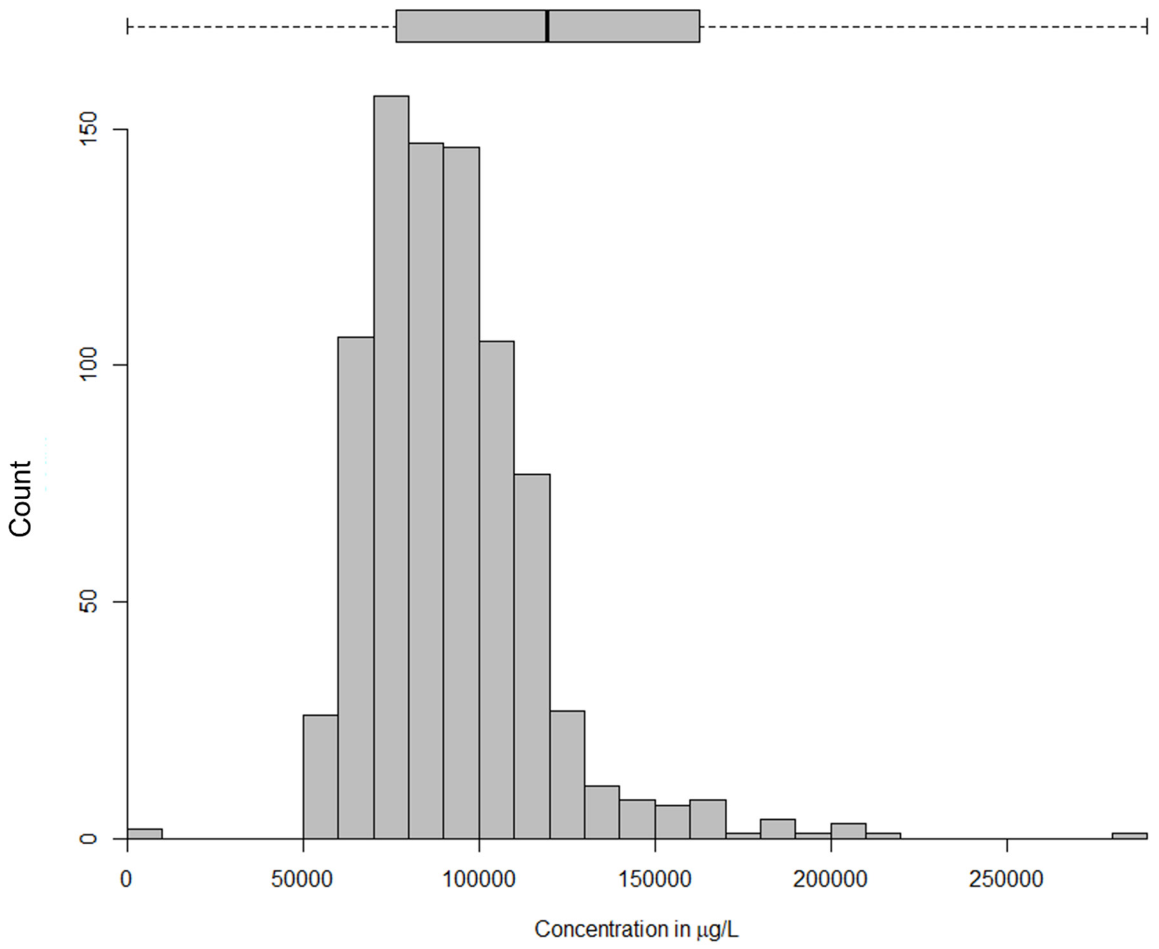

**Supplementary Figure S2.** Directed Acyclic Graph to identify potential confounders and mediators in the relation of propionic acid in serum to cognitive decline

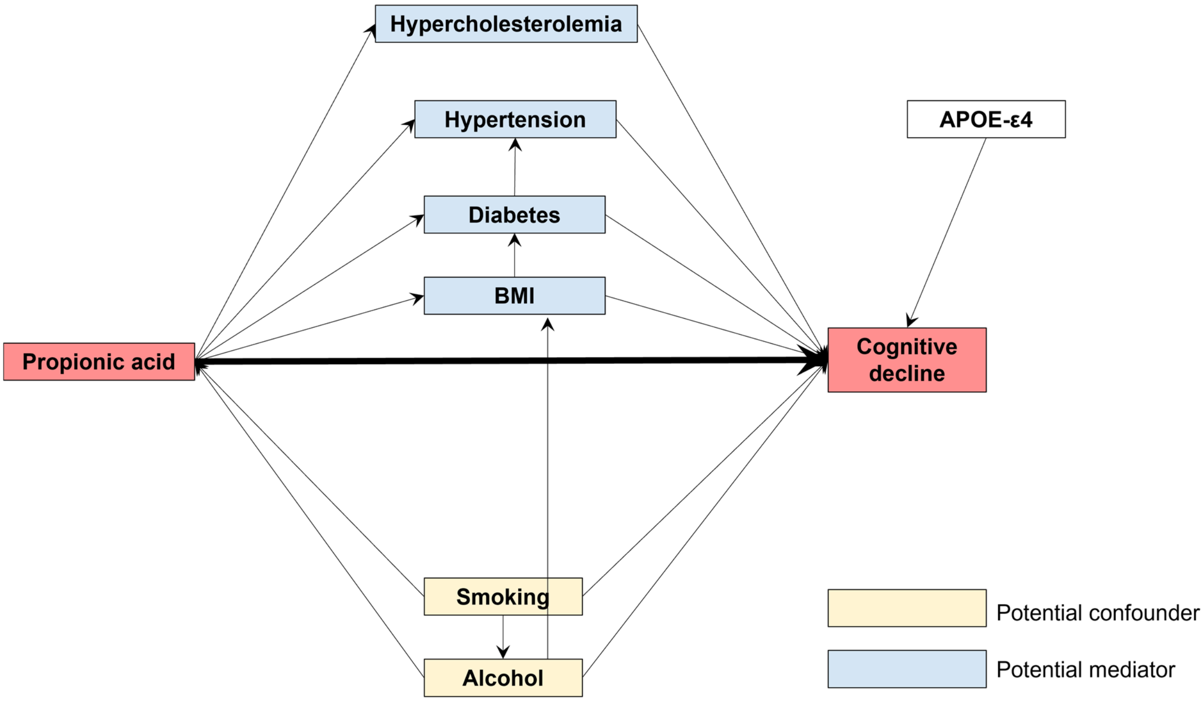


Footnote: A directed acyclic graph (DAG) was implemented using DAGitty software (version 3.0) to identify the factors of adjustment in the model estimating the association between propionic acid in serum and cognitive decline. Covariates and their potential role in the relation of propionic acid to cognitive decline (beyond the matching variables age, sex and education) were selected from the literature.

**Supplementary Figure S3.** Counterfactual mediation analysis for matched data to estimate the mediating effect of hypertension, hypercholesterolemia and diabetes in the relation of propionic acid in serum to the odds of cognitive decline over 12 years in the pooled sample (n=838)

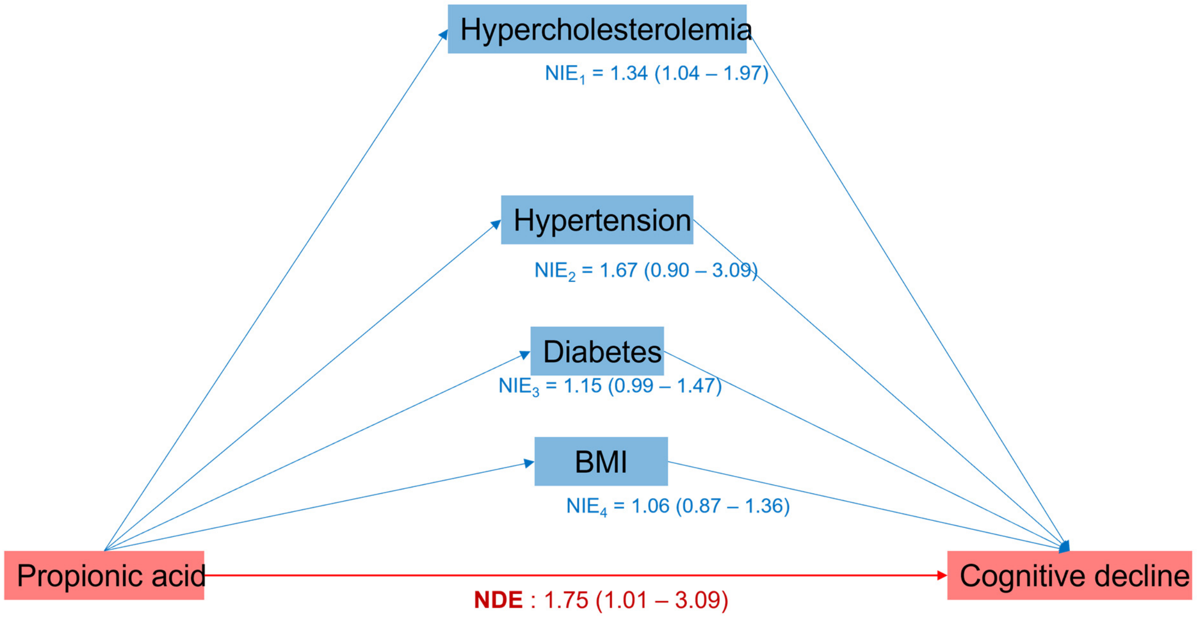


Footnote: A counterfactual mediation analysis for matched data[1,2] was run independently for each of the four potential mediators using conditional logistic regression models adjusted for confounders (alcohol consumption and smoking) and other potential mediators (BMI, hypercholesterolemia, hypertension or diabetes). Propionic acid and mediators were modeled as a binary variables (concentration ≥75th vs <25th percentiles for propionic acid; ≥30 kg/m² vs < 30 kg/m² for BMI, yes/no for hypertension, hypercholesterolemia and diabetes).

Note that there is no method available yet, to our knowledge, to assess in a unique analysis multiple mediation through potentially correlated mediators in matched case-control studies; here, we adapted the method by Kim et al[2] to several mediators, modeled non-concomitantly. In counterfactual approaches, two assumptions about unmeasured confounders must hold: (i) There is no mediator – outcome confounder affected by exposure, and (ii) no exposure-mediator interaction. There was no statistically significant interaction between propionic acid (the exposure) and any of the 4 mediators on the odds of cognitive decline. In each model, the total effect of propionic acid on cognitive decline was decomposed into a natural direct effect (NDE, red arrow) and a natural indirect effect ($NIE_1$, $NIE_2$, $NIE_3$ and $NIE_4$, blue arrows) through the mediator. NDE is interpreted as the effect of propionic acid on cognitive decline when the path between each mediator and cognitive decline is blocked. NIE represents the impact of propionic acid on cognitive decline when the value of the mediator changes.

**References**


1. VanderWeele, T. J. Mediation Analysis: A Practitioner's Guide. *Annu. Rev. Public Health* **2016**, *37*, 17–32
2. Kim, Y. M. *et al.* Causal mediation analysis in nested case-control studies using conditional logistic regression. *Biom. J.* **2020**, *62*, 1939–1959
